# Two Distinct Mechanisms Underlying γδ T Cell-Mediated Regulation of Collagen Type I in Lung Fibroblasts

**DOI:** 10.3390/cells11182816

**Published:** 2022-09-09

**Authors:** Daisuke Okuno, Noriho Sakamoto, Yoshiko Akiyama, Takatomo Tokito, Atsuko Hara, Takashi Kido, Hiroshi Ishimoto, Yuji Ishimatsu, Mohammed S. O. Tagod, Haruki Okamura, Yoshimasa Tanaka, Hiroshi Mukae

**Affiliations:** 1 Department of Respiratory Medicine, Graduate School of Biomedical Sciences, Nagasaki University, Nagasaki 852-8501, Japan; 2 Department of Nursing, Graduate School of Biomedical Sciences, Nagasaki University, Nagasaki 852-8520, Japan; 3 Center for Medical Innovation, Nagasaki University, Nagasaki 852-8588, Japan; 4 Laboratory of Tumor Immunology and Cell Therapy, Hyogo College of Medicine, Nishinomiya 663-8501, Japan

**Keywords:** γδ T cell, idiopathic pulmonary fibrosis, HMBPP, interferon-γ, interleukin-18

## Abstract

Idiopathic pulmonary fibrosis is a chronic intractable lung disease, leading to respiratory failure and death. Although anti-fibrotic agents delay disease progression, they are not considered curative treatments, and alternative modalities have attracted attention. We examined the effect of human γδ T cells on collagen type I in lung fibroblasts. Collagen type I was markedly reduced in a γδ T cell number-dependent manner following treatment with γδ T cells expanded with tetrakis-pivaloxymethyl 2-(thiazole-2-ylamino) ethylidene-1,1-bisphosphonate (PTA) and interleukin-2. Collagen type I levels remained unchanged on addition of γδ T cells to the culture system through a trans-well culture membrane, suggesting that cell–cell contact is essential for reducing its levels in lung fibroblasts. Re-stimulating γδ T cells with (E)-4-hydroxy-3-methylbut-2-enyl diphosphate (HMBPP) reduced collagen type I levels without cell–cell contact, indicating the existence of HMBPP-induced soluble anti-fibrotic factors in γδ T cells. Adding anti-interferon-γ (IFN-γ)-neutralizing mAb restored collagen type I levels, demonstrating that human γδ T cell-derived IFN-γ reduces collagen type I levels. Conversely, interleukin-18 augmented γδ T cell-induced suppression of collagen type I. Therefore, human γδ T cells reduce collagen levels in lung fibroblasts via two distinct mechanisms; adoptive γδ T cell transfer is potentially a new therapeutic candidate.

## 1. Introduction

Idiopathic pulmonary fibrosis (IPF) is a chronic progressive fibrotic lung disease of unknown etiology that is characterized by interstitial fibrosis and irreversible destruction of the lung architecture [1]. IPF prognosis is poor in the absence of treatment, and the five-year survival rate is lower than that of certain cancers [2]. Although antifibrotic drugs such as nintedanib and pirfenidone delay disease progression and improve patient prognoses, these small molecule-based therapies fail to achieve curative effects [3,4]. Developing alternative modalities for IPF treatment is, therefore, imperative.

Fibrosis is caused by repetitive epithelial injury followed by exaggerated and dysregulated wound repair, leading to the accumulation of extracellular matrix and resident lung fibroblasts and remodeling of the lung architecture. Myofibroblasts are the primary collagen-producing cells and, thus, play a key role as cellular mediators in the development of fibrosis [5], indicating that the resident cell subsets are a promising target for the development of antifibrotic therapy.

Both innate and adaptive immune responses are induced in fibrotic diseases, leading to the activation of a variety of inflammatory immune cells, including neutrophils, macrophages, and lymphocytes [5,6,7]. The adaptive immune system consists of B cells and T cells that contain antigen receptors generated through gene recombination. T cells are divided into αβ and γδ T cells. The majority of human peripheral blood T cells express αβ T cell receptors (TCRs) and recognize antigenic peptides presented by major histocompatibility complex class I or class II molecules with the help of CD8 or CD4 markers, whereas most other T cells express γδ TCRs, particularly Vγ9Vδ2 (also called Vγ2Vδ2, depending on nomenclature)-containing TCRs that recognize foreign (E)-4-hydroxy-3-methylbut-2-enyl diphosphate (HMBPP, a microbial γδ T cell antigen), endogenous isopentenyl diphosphate (IPP), and dimethylallyl diphosphate (DMAPP), depending on butyrophilin 3A1/2A1 levels.

γδ T cells are a unique subset of lymphocytes involved in both infection immunity and tumor immunity, including immune responses to inhaled microbial pathogens and allergens [8,9,10]. Certain murine γδ T cell subsets play a role in the prevention of fibrotic diseases; however, little is known about the role of human γδ T cells in lung fibrotic diseases [11,12,13,14,15,16], partly because there is no murine equivalent of Vγ9Vδ2-bearing γδ T cells. Human γδ T cells regulate fibrosis in systemic sclerosis (SSc) [17,18,19,20,21,22]; however, the precise mechanism of action has not yet been elucidated.

Peripheral blood mononuclear cell supernatants treated with IPP inhibited procollagen secretion in fibroblasts [17]. Conversely, γδ T cell supernatants enhanced collagen synthesis and promoted the proliferation of human skin fibroblasts [18]. Additionally, fibroblasts co-cultured with γδ T cells exhibited elevated pro-α1 (I) collagen (COL1A1) and pro-α2 (I) collagen (COL1A2) mRNA levels compared with those co-cultured with non-γδ T cells [19].

These contradictory results may stem from impurities in human γδ T cell preparations. In this study, we aimed to develop a novel cell-based therapy for IPF. We employed a γδ T cell culture system using tetrakis-pivaloxymethyl 2-(thiazole-2-ylamino) ethylidene-1,1-bisphosphonate (PTA), a nitrogen-containing bisphosphonate prodrug and an inhibitor of farnesyl diphosphate synthase (FDPS) [23,24] that allowed us to obtain a large number of purified γδ T cells in 10–11 days [24,25]. Then, we examined how γδ T cells affect the regulation of collagen type I in lung fibroblasts.

## 2. Materials and Methods

### 2.1. Isolation of γδ T Cells and Culture of Fibroblasts

This study was approved by the Institutional Review Board of the Nagasaki University Hospital. Peripheral blood samples were obtained from healthy adult volunteers and patients with IPF. Written informed consent was obtained from all the participants. Heparin sodium (Mochida Pharmaceutical, Co., Ltd., Shinjuku-ku, Tokyo, Japan; 1/100 volume) was added to blood samples diluted with equal volumes of Dulbecco’s phosphate-buffered saline (PBS) (-) (Nissui Pharmaceutical Co., Ltd., Minato-ku, Tokyo, Japan). Diluted blood samples (20 mL) were loaded onto 20 mL Ficoll-Paque^TM^ PLUS (GE Healthcare BioSciences AB, Chicago, IL, USA) in 50 mL conical tubes (Corning Inc., Corning, NY, USA) and centrifuged at 600× *g* at 25 °C for 30 min. The fluffy layer was then transferred into a new 50 mL conical tube and diluted with 2.5 volumes of PBS (-). The peripheral blood mononuclear cell (PBMC) suspension was centrifuged at 900× *g* at 4 °C for 10 min. After the supernatant was aspirated, cell pellets were dispersed by tapping and then resuspended in 13 mL PBS (-). The cell suspension was centrifuged at 600× *g* at 4 °C for 5 min, and the supernatant was aspirated. Subsequently, the cell pellets were dispersed by tapping and resuspended in 7 mL of Yssel’s medium consisting of Iscove’s modified Dulbecco’s medium (Thermo Fisher Scientific, Waltham, MA, USA) supplemented with 10% human AB serum (Cosmo Bio Co., Ltd., Koto-ku, Tokyo, Japan), 3.6 × 10^−2^ M NaHCO_3_ (Nacalai Tesque Inc., Nakagyo-ku, Kyoto, Japan), 3.3 × 10^−5^ M 2-aminoethanol (Nacalai Tesque Inc., Kyoto, Japan), 40 mg/L transferin apo form (Nacalai Tesque Inc., Kyoto, Japan), 5 mg/L human recombinant insulin (Merck & Co., Inc., Darmstadt, Hesse, Germany), 2 mg/L linoleic acid (Merck & Co., Inc., Darmstadt, Germany), 2 mg/L oleic acid (Merck & Co., Inc., Darmstadt, Germany), 2 mg/mL palmitic acid (Merck & Co., Inc., Darmstadt, Germany), 100 μg/mL streptomycin, and 100 U/mL penicillin. The PBMC suspension (1.5 mL each, 1–2.5 × 10^6^ cells/mL) was dispensed into the wells of a 24-well plate (Corning Inc., Corning, NY, USA). A PTA stock solution (1.5 μL, 1 mM) in dimethyl sulfoxide at a final concentration of 1 μM was added to each well. The plate was incubated at 37 °C and 5% CO_2_ for 24 h. Interleukin-2 (IL-2, Shionogi Pharmaceutical Co., Ltd., Chuo-ku, Osaka, Japan) was added to each well every day from Day 1 to Day 5 to obtain a final concentration of 100 U/mL. On Day 6, 1.5 mL of Yssel’s medium was added to the wells, and the diluted cell suspension was mixed by pipetting. Half of the suspension was transferred into a new well, and 100 U/mL of IL-2 was added. On Day 7, 1.5 mL of complete RPMI 1640 medium (Merck & Co., Inc., Darmstadt, Germany) supplemented with 10% fetal calf serum (FCS, Merck & Co., Inc., Darmstadt, Germany), 10^−5^ M of 2-mercaptoethanol (Wako Pure Chemical Industries, Ltd., Osaka, Japan), 100 μg/mL streptomycin (Meiji Seika Pharma Co., Ltd., Tokyo, Japan), and 100 U/mL penicillin (Meiji Seika Pharma Co., Ltd.) was added to each well. Each cell suspension was split into two wells, and IL-2 was added at a final concentration of 100 U/mL. Vγ9Vδ2-positive γδ T cells were expanded using complete RPMI 1640 medium plus 100 U/mL of IL-2 by Day 11, harvested by centrifuging at 600× *g* at 4 °C for 5 min, resuspended in cryopreservation media, dispensed into cryovials, incubated overnight at −80 °C, and then stored in liquid nitrogen until use. Cells collected on Day 0 and Day 11 were stained with 3 μL of phycoerythrin (PE)-conjugated anti-CD3 mAb (BD Biosciences, San Diego, CA), NKG2D, DNAM-1, FasL, or tumor necrosis factor-related apoptosis-inducing ligand (TRAIL) mAb (BioLegend, San Diego, CA), and FITC-conjugated anti-TCR Vδ2 mAb (BD Biosciences) on ice for 15 min in a 96-well round-bottom plate. After three washes using 200 μL PBS (-) supplemented with 2% FCS, the cells were resuspended in 200 μL of PBS (-)/2% FCS and analyzed using a FACSLyric flow cytometer (BD Biosciences). The cell population was visualized using FlowJo software ver. 10 (FlowJo LLC, Ashland, OR). Diseased human lung fibroblasts derived from a patient with IPF (DHLF, Lonza Walkersville, Inc., Basel, Switzerland) were cultured in complete RPMI 1640 medium in 75 cm^2^ flasks (Corning Inc., Corning, NY, USA) at 37 °C and 5% CO_2_. Assays were conducted using cells with low passage numbers (passages 3–7) to avoid altered morphologies, growth rates, and responses to stimuli.

### 2.2. Co-Culture of Lung Fibroblasts with γδ T Cells

DHLF cells (2.5 × 10^4^ cells in 1 mL of complete RPMI 1640 medium) were cultured overnight in a Lab-Tek chamber slide (Thermo Fisher Scientific, Waltham, MA, USA). After the supernatant was aspirated, 1 mL of thawed γδ T cells (2 × 10^6^) or complete RPMI 1640 medium was added to the chamber slide, and the mixture was co-cultured at 37 °C and 5% CO_2_ for 2 days. Collagen type I in DHLF cells was analyzed via immunocytochemistry. Western blotting and real-time reverse transcription polymerase chain reaction (real-time RT-PCR) assays were performed to confirm the collagen expression levels. Briefly, the DHLF cells (1.5 × 10^5^ cells in 2 mL of complete RPMI 1640 medium) were seeded in a 6-well plate (Corning Inc., Corning, NY, USA). Either 1 μL of complete RPMI 1640 medium or 1 μL of transforming growth factor-β1 (TGF-β) (final concentration: 5 ng/mL) was then added. The plate was incubated overnight at 37 °C and 5% CO_2_, the supernatant was aspirated, and 2 mL of PBS (-) was added to the well. After aspirating the supernatant, 2 mL of serially diluted PTA-expanded γδ T cells (6.25 × 10^5^, 1.25 × 10^6^, 2.5 × 10^6^, 5 × 10^6^, or 1 × 10^7^ cells for Western blot assay; 2 × 10^6^ or 5 × 10^6^ cells for RT-PCR) or complete RPMI 1640 medium was added, and the plate was incubated for 2 days at 37 °C and 5% CO_2_. The supernatant was aspirated, and the cells were washed twice with 2 mL of PBS (-) to remove γδ T cells. For Western blot analysis, 100 μL of RIPA buffer containing 1% protease/phosphatase inhibitor (Thermo Fisher Scientific, Waltham, MA, USA) was added to each well, and the plate was then incubated on ice for 5 min. Cell lysates were transferred into tubes using a Coster cell scraper (Corning Inc., Corning, NY, USA) and centrifuged at 14,000× *g* for 15 min at 4 °C. The supernatants were then transferred into new tubes and stored at −80 ℃ until use. Collagen type I in DHLF cells was analyzed via Western blotting. To conduct real-time RT-PCR assays, cells were dissolved in 350 μL of lysis buffer (RLT lysis buffer containing 1% β-mercaptoethanol) using a Coster cell scraper (Corning Inc., Corning, NY, USA), transferred into safe-lock tubes (Eppendorf, Hamburg, Germany), and stored at −80 ℃ until use. Total RNA was prepared using an RNeasy Plus mini kit (QIAgen, Valencia, CA), and COL1A1 and COL1A2 mRNA expression was quantified using the standard protocol. To analyze γδ T cell cytotoxicity, DHLF cells (2 × 10^4^) treated with TGF-β (5 ng/mL) or complete RPMI 1640 medium were cultured overnight in a 96-well flat-bottom plate (Corning Inc., Corning, NY, USA) at 37 ℃ and 5% CO_2_. The supernatants were then aspirated and the cells were washed with 200 μL of PBS (-). After removing the supernatant, 200 μL of γδ T cells at effector-to-target cell ratios (E/T ratios) of 0, 6.25:1, 12.5:1, 25:1, 50:1, and 100:1 were added into the wells, and the plate was incubated for 24 h at 37 ℃ and 5% CO_2_. The supernatants were then aspirated, and the cells were washed three times with 200 μL of complete RPMI 1640 medium. A total of 100 μL of CellTiter-Glo^®^ reagent (PerkinElmer Inc., Ealtham, MA, USA) was subsequently added into each well, and cell lysates were transferred into a 96-well OptiPlate (PerkinElmer Inc.). Luminescence was measured using an ARVO multiplate reader (PerkinElmer Inc.).

### 2.3. Co-Culture of Lung Fibroblasts and γδ T Cells through a Membrane Insert

DHLF cells (1.5 × 10^5^ cells in 2 mL complete RPMI 1640 medium) were placed in a 6-well plate, combined with either 1 μL of TGF-β (final concentration 5 ng/mL) or 1 μL of complete RPMI 1640 medium, and incubated overnight at 37 °C and 5% CO_2_. The supernatant was aspirated, and 2 mL of PBS (-) was added. Supernatants were then removed, and 1.5 mL of complete RPMI 1640 medium was added into the wells. Next, cell culture inserts (0.4 μm pore size; polyethylene terephthalate membrane, Corning Inc., Corning, NY, USA) were placed into the 6-well plate, and 1 mL of γδ T cells (6.25 × 10^5^, 1.25 × 10^6^, 2.5 × 10^6^, 5.0 × 10^6^, or 1.0 × 10^7^ cells) or complete RPMI 1640 medium was added. The plate was incubated for 2 days at 37 °C and 5% CO_2_. After this, the cell culture inserts were removed, the supernatants were aspirated, and the cells were washed with PBS (-). Cell lysates were dissolved in RIPA buffer as described earlier, and collagen type I was analyzed using Western blot assay.

### 2.4. Flow Cytometric Analysis of LFA-1 Expression on γδ T Cells

γδ T cell suspensions were placed in a 96-well round-bottom plate and centrifuged at 600× *g* and 4 °C for 2 min. The supernatants were then aspirated, cell pellets were resuspended in 50 μL of PBS/2% FCS, and 2 μg/mL of mouse anti-human lymphocyte function-associated antigen-1 (LFA-1, or CD11a/CD18) mAb (BioXcell, West Lebanon, NH, USA) was added. The plate was placed on ice for 30 min, and the cells were washed three times with 200 μL of PBS/2% FCS and resuspended in 50 μL of PBS/2% FCS. The cells were subsequently placed on ice for 15 min and stained with fluorescein isothiocyanate (FITC)-conjugated anti-TCR Vδ2 mAb and R-phycoerythrin (RPE)-conjugated anti-mouse Ig (Agilent Technologies, Inc., Santa Clara, CA). The cells were then washed three times with 200 μL of PBS (-) and resuspended in 200 μL of PBS (-). Stained cells were analyzed using a FACSLyric flow cytometer, and the cell population was visualized using FlowJo software ver. 10.

### 2.5. Effect of Anti-LFA-1 mAb on Collagen Type I in Lung Fibroblasts Co-Cultured with γδ T Cells

DHLF cells (1.5 × 10^5^) in 2 mL of complete RPMI 1640 medium were placed in a 6-well plate and incubated overnight at 37 °C and 5% CO_2_. The supernatant was aspirated, and 2 mL of γδ T cell suspension (5 × 10^6^ cells) or complete RPMI 1640 medium was added. Anti-LFA1 antibody was added to obtain a final concentration of 2 μg/mL, and the plate was incubated for 2 days at 37 °C and 5% CO_2_. The cells were washed twice with 2 mL of PBS (-) and then dissolved in RIPA buffer, as described earlier. Collagen type I and αSMA protein expression levels in DHLF cells were analyzed via Western blot assays.

### 2.6. Co-Culturing Lung Fibroblasts and γδ T Cells Stimulated with HMBPP

DHLF cells (2.5 × 10^4^) were cultured in 1 mL of complete RPMI 1640 medium overnight in a Lab-Tek chamber slide at 37 ℃ and 5% CO_2_. The supernatant was removed, and 1 mL of γδ T cell suspension (5 × 10^3^ cells) or complete RPMI 1640 medium was added to the chamber slide. Then, HMBPP, which was synthesized in our laboratory, was added to the chamber to obtain a final concentration of 10 μM. The plate was incubated for 2 days at 37 ℃ and 5% CO_2_, and collagen type I levels in DHLF cells were examined via immunocytochemistry. For Western blot analysis, DHLF cells (1.5 × 10^5^) were placed in a 6-well plate and incubated overnight at 37 ℃ and 5% CO_2_. After the supernatant was removed, γδ T cell suspensions (0, 0.15 × 10^5^, 0.75 × 10^5^, or 1.5 × 10^5^ cells) and HMBPP (final concentration of 10 μM) were added, and the plate was incubated at 37 ℃ and 5% CO_2_ for 2 days. DHLF cells were then washed twice with 2 mL of PBS (-), dissolved in RIPA buffer (as described earlier), and collagen type I and αSMA protein expression levels were examined using Western blot analysis.

To examine the effect of HMBPP on the γδ T cell-mediated modulation of collagen levels in fibroblasts, DHLF cells (1.5 × 10^5^) treated with TGF-β (5 ng/mL) or RPMI 1640 (negative control) were incubated overnight in a 6-well plate at 37 °C and 5% CO_2_. The cells were washed with PBS (-), and then 1.5 mL of complete RPMI 1640 was added and culture membrane inserts were placed in the wells. γδ T cells (5.0 × 10^6^) or 1 mL of complete RPMI 1640 medium was added to the culture membrane inserts, and HMBPP (final concentrations of 0, 0.1, 1, and 10 μM) was added. After incubation for 2 days at 37 °C and 5% CO_2_, the culture inserts were removed and DHLF cells were dissolved in lysis buffer, as described earlier. Then, the collagen levels were determined via Western blot analysis.

### 2.7. Effect of γδ T Cell Supernatants on Lung Fibroblasts

γδ T cells (5 × 10^6^) in 1.5 mL of complete RPMI 1640 medium were cultured overnight at 37 °C and 5% CO_2_ in a 24-well plate (Corning Inc., Corning, NY, USA) in the presence or absence of HMBPP (final concentration of 10 μM). The supernatant was then transferred into conical tubes and centrifuged at 600× *g* and 4 °C for 5 min; subsequently, the supernatant was transferred into new conical tubes and stored at −80 °C until use. DHLF cells (2.5 × 10^4^) were cultured overnight in a Lab-Tek chamber slide, and the supernatant was then aspirated. The γδ T cell culture supernatant (500 μL) was thawed and combined with 500 μL of complete RPMI 1640 medium on the chamber slide. Complete RPMI 1640 medium (1 mL) was added to the chamber slide as a control. The slide was incubated for 2 days at 37 °C and 5% CO_2_, and collagen type I expression was then determined via immunocytochemistry.

To examine the effect of cytokines in γδ T cell culture supernatants on the modulation of collagen levels, DHLF cells (1.5 × 10^5^) were cultured overnight in a 6-well plate at 37 °C and 5% CO_2_. γδ T cell culture supernatants were thawed and transferred into conical tubes. Anti-IFN-γ antibodies (Biolegend) or anti-tumor necrosis factor-α (TNF-α) antibodies (Biolegend) were added to obtain concentrations of 0, 0.1, 1, or 10 μg/mL. The tubes were placed at room temperature for 15 min. DHLF culture supernatants were aspirated, and 1 mL of complete RPMI 1640 medium was added to the chamber slide. A mixture of γδ T cell culture supernatant and serially diluted antibody was then added to the slide. The complete RPMI 1640 medium (2 mL) was used as a control, and HMBPP (final concentration of 10 μM) was added. The plate was incubated for 2 days at 37 °C and 5% CO_2_, and the DHLF cells were dissolved in lysis buffer as described earlier and analyzed via Western blotting.

To examine the effect of IFN-γ on the modulation of collagen levels, γδ T cells (5 × 10^6^) were cultured overnight in the presence or absence of HMBPP (final concentration of 0.1 nM) and interleukin-18 (IL-18; final concentration of 100 ng/mL) in a 24-well plate at 37 °C and 5% CO_2_. The cells were centrifuged at 600× *g* and 4 °C for 5 min, and the supernatants were transferred into conical tubes and stored at −80 ℃ until use. The culture supernatants were removed after incubating DHLF cells (1.5 × 10^5^) overnight in a 6-well plate at 37 °C and 5% CO_2_. One milliliter of thawed γδ T cell culture supernatant and 1 mL of complete RPMI 1640 medium were added to the wells. The complete RPMI 1640 medium (2 mL) in the presence or absence of HMBPP (final concentration of 0.1 nM) and IL-18 (final concentration of 100 ng/mL) was added to the wells as a control. The plate was then incubated for 2 days at 37 ℃ and 5% CO_2_, after which the DHLF cells were dissolved in lysis buffer, and collagen expression was determined via Western blot assay.

To further examine the effect of IFN-γ on the modulation of collagen levels, thawed γδ T cell culture supernatants were treated with anti-IFN-γ antibodies (0, 1, or 10 μg/mL) at room temperature for 15 min. DHLF cells were then cultured in a 6-well plate, as described earlier, and the supernatants were aspirated. Then, 1 mL of γδ T cell culture supernatant in the presence or absence of anti-IFN-γ antibody and 1 mL of complete RPMI 1640 were added to the wells. Complete RPMI 1640 (2 mL) in the presence or absence of HMBPP (0.1 nM) and IL-18 (100 ng/mL) was added to the wells as a control. The plate was incubated for 2 days, and DHLF cells were analyzed for collagen expression levels via Western blot analysis.

### 2.8. IFN-γ and TNF-α Enzyme-Linked Immunosorbent Assay

To determine IFN-γ and TNF-α secretion from γδ T cells in response to HMBPP, γδ T cells (4 × 10^5^ cells in 198 μL of complete RPMI 1640 medium) were added to a 96-well flat-bottom plate, and 2 μL of HMBPP was added to obtain final concentrations of 0.01 nM, 0.1 nM, 1 nM, 10 nM, 100 nM, 1 μM, or 10 μM. The plate was incubated for 24 h at 37 °C and 5% CO_2_. The cell suspension was mixed well, and the plate was centrifuged at 600× *g* and 4 °C for 2 min. The supernatant was then transferred into a 96-well round-bottom plate, and the plate was stored at −80 °C for 24 h. The samples were then thawed, and IFN-γ and TNF-α levels were analyzed via enzyme-linked immunosorbent assay (ELISA, Peprotech, Rocky Hill, NJ), according to the manufacturer’s instructions. To measure IFN-γ secretion from γδ T cells in response to HMBPP and IL-18, γδ T cells (4 × 10^5^) were resuspended in 196 μL of complete RPMI 1640 medium in a 96-well flat-bottom plate, and 2 μL of HMBPP at final concentrations of 1, 10, 100, 1, or 10 nM and 2 μL of IL-18 (final concentration of 100 ng/mL) or complete RPMI 1640 medium were added. After incubation for 24 h at 37 °C and 5% CO_2_, the supernatants were transferred into a 96-well round-bottom plate and stored at −80 °C.

### 2.9. Effect of Human γδ T Cells on Mouse Lung Fibroblasts

Mouse lung fibroblasts (Mlg) (1.5 × 10^5^ cells) were cultured overnight in a 6-well plate at 37 °C and 5% CO_2_. Culture supernatants were removed and combined with human γδ T cells (5 × 10^6^ cells) directly or through a culture membrane insert. The effect of HMBPP (10 μM) was examined by adding HMBPP through the culture membrane insert. The plate was incubated for 2 days, and collagen levels in Mlg were measured via Western blot analysis.

### 2.10. Immunocytochemistry

To stain cells, culture supernatants were removed from chamber slides, and the cells were washed three times with 0.5 mL of PBS (-). Phosphate buffer containing 4% paraformaldehyde (0.5 mL, Fujifilm Wako Pure Chemical Corp. Chuo-ku, Osaka, Japan) was added to the chamber slide, and the slide was placed at room temperature for 10 min. The solution was then aspirated, and the chamber slide was washed three times with 0.5 mL of PBS (-). Hydrogen peroxide solution (0.5 mL, 0.3% in methanol, Fujifilm Wako Pure Chemical Corp., Osaka, Japan) was added to the chamber slide, and the slide was incubated at room temperature for 20 min. The solution was then aspirated, and the cells were washed three times with 0.5 mL PBS (-). Anti-collagen type I polyclonal antibody (Thermo Fisher Scientific, Waltham, MA, USA) was diluted at a ratio of 1:500 with 0.1% bovine serum albumin solution in PBS (-), and 200 μL of this diluted solution was added to the chamber slide, which was incubated overnight at 4 °C. Normal rabbit IgG (200 μL, Santa Cruz Biotechnology Inc., Santa Cruz, CA) was used as a negative control. The antibody solution was aspirated, and 0.5 mL of PBS (-) was added to the chamber slide. The chamber slide was then incubated for 3 min, and the obtained solution was discarded. This step was repeated two more times, following which two drops of Histofine Simple Stain MAX-PO(R) (Nichirei Co., Tokyo, Japan) were added to the chamber slide, and the slide was incubated for 30 min at room temperature. The solution was aspirated, and the chamber slide was washed four times with 0.5 mL of PBS (-) every 3 min. The coloring reagent (200 μL) containing 5 mg of 3,3′-diaminobenzidine tetrahydrochloride (Fujifilm Wako Pure Chemical Corp., Osaka, Japan), 10 mL of 50 mM Tris buffer (Sigma-Aldrich, St Louis, MO, USA), and 10 μL of 30% hydrogen peroxide solution (Fujifilm Wako Pure Chemical Corp., Osaka, Japan) was added to the slide, which was then incubated at room temperature for 10 min. The solution was subsequently aspirated, and the chamber slide was immersed in a tray of distilled water for 2 min and then in another tray of distilled water for 2 s. The water was aspirated, and 300 μL of Mayer’s hematoxylin solution (Fujifilm Wako Pure Chemical Corp., Osaka, Japan) was added to the chamber slide. After incubation for 10 min, the solution was removed, and the slide was immersed in a tray of distilled water for 2 min and then in another tray of distilled water for 2 s. Finally, the chamber slide was air-dried and observed under a microscope (ECLIPSE Ci, Nikon Instruments Inc., Tokyo, Japan).

### 2.11. Western Blot Assay

Protein concentrations in cell lysates were determined using a BCA protein assay kit (Thermo Fisher Scientific, Waltham, MA, USA), according to the manufacturer’s instructions, and optical density was measured at 540 nm using a multiplate reader (Thermo Fisher Scientific, Waltham, MA, USA). Cell lysates (30 μg in 30 μL of RIPA buffer) were diluted with equal volumes of Laemmli sample buffer (Bio-Rad, Hercules, CA, USA) containing 5% 2-mercaptoethanol (Fujifilm Wako Pure Chemical Corp., Osaka, Japan) to obtain a final protein concentration of 5 μg/10 μL. The samples were then vortexed thoroughly, placed on a heating block (AGC Techno Glass Co., Ltd., Haibara, Shizuoka, Japan) set at 95 °C for 5 min, vortexed once more, and placed on ice for 10 min. Samples (10 μL) were loaded into lanes on a 4–15% Mini-PROTEAN^®^ TGX™ Gel (Bio-Rad, Hercules, CA, USA) and run at 200 V and 40 mA for 30 min. The protein bands were transferred onto a membrane for 7 min using a Trans-Blot^®^ Turbo^TM^ Transfer System (MW-1.3A, Bio-Rad, Hercules, CA, USA). The membrane was then immersed in a plastic container with 20 mL blocking buffer containing 5% BSA in Tris-buffered saline with Tween 20 (TBST, 1 tablet in 500 mL of distilled water, Takara Bio Inc., Kusatsu, Shiga, Japan) and incubated for 1 h. After the blocking buffer was removed, the membrane was washed with TBST, cut into pieces, and incubated overnight at 4 °C in a Hybri-Bag (Cosmo Bio Co., Ltd., Koto-ku, Tokyo, Japan) containing rabbit anti-collagen I polyclonal antibody (1/5000 dilution, Thermo Fisher Scientific, Waltham, MA, USA) or mouse anti-GAPDH polyclonal antibody (1/1000 dilution, Thermo Fisher Scientific, Waltham, MA, USA). The membrane was subsequently washed three times with 20 mL of TBST for 10 min and then treated with horseradish peroxidase (HRP)-labeled secondary antibody, anti-rabbit IgG antibody (1/5000 dilution, Abcam, Cambridge, UK), or anti-mouse IgG antibody (1/1000 dilution, Biotechne, Minneapolis, MN, USA) at room temperature for 1 h. The membranes (for collagen type I) were washed three times with 20 mL of TBST for 10 min and treated with Clarity^TM^ Western ECL Substrate (Bio-Rad, Hercules, CA, USA). Membrane luminescence was monitored using a ChemiDoc™ MP Imaging System (Bio-Rad, Hercules, CA, USA). The membranes (for GAPDH) were washed with TBST for 15 min and treated with Restore^TM^ PLUS Western blot stripping buffer (Thermo Fisher Scientific, Waltham, MA, USA) for 15 min and then with TBST for 15 min before the treatment with blocking buffer for 1 h. The membranes were then placed in a Hybri-Bag containing anti-αSMA antibody (1/1000 dilution, Abcam) and incubated overnight at 4 °C. Subsequently, the membranes were washed three times with TBST for 10 min and incubated at room temperature for 1 h with the secondary antibody (anti-rabbit IgG antibody). The membranes were subsequently washed three times with TBST for 10 min, and luminescence was measured as described earlier. Relative collagen and αSMA expression intensities were calculated by normalization to GAPDH levels using Image Lab™ software ver. 5.2.1 (Bio-Rad, Hercules, CA, USA).

### 2.12. Real-Time RT-PCR

Cells were dissolved in lysis buffer and transferred to a microcentrifuge tube. The tube was vortexed three times for 10 s and spun down. Total RNA was extracted from the supernatant using a QIAcube kit (QIAGEN, Hilden, Dusseldorf, Germany), according to the manufacturer’s instructions, and quantified using a Nano Drop spectrophotometer (Thermo Fisher Scientific, Waltham, MA, USA). Purified RNA was diluted with diethylpyrocarbonate-treated water (part of the SuperScript^®^Ⅲ First-Strand Synthesis System (SS III kit), Thermo Fisher Scientific, Waltham, MA, USA) to obtain a concentration of 2 μg/8 μL, and transferred into PCR tubes (NIPPON Genetics Co., Ltd., Tokyo, Japan) on ice. Two microliters of oligo(dT)20 (50 μM, Thermo Fisher Scientific, Waltham, MA, USA) and deoxyribonucleotides (10 mM, part of the SS III kit) were added to the diluted RNA (8 μL), and this mixture was placed on ice. The samples were then centrifuged at 14,000 rpm and 4 °C for 7 s and incubated at 65 ℃ for 5 min on a T100^TM^ thermal cycler (Bio-Rad, Hercules, CA, USA). The samples were incubated on ice for 1 min, followed by the addition of 10 μL of a reagent consisting of 10× reverse transcription buffer (2.25 μL), 25 mM MgCl (4.5 μL), 0.1 M dithiothreitol (2.25 μL), RNaseOUT^TM^ (40 U/μL, 1.125 μL), and SuperScript^®^ III RT (200 U/μL, 1.125 μL; part of the SS III kit). The samples were incubated at 50 °C for 50 min and then at 85 °C for 5 min on a thermal cycler (Bio-Rad, Hercules, CA, USA). Subsequently, the samples were centrifuged at 14,000 rpm for 7 s at 4 °C. RNase H (1 μL, part of the SS III kit) was added, and the tubes were incubated at 37 ℃ for 20 min on the thermal cycler. The synthesized cDNA was diluted 2.5-fold with nuclease-free water. Then, the diluted cDNA (2.2 μL) was transferred into a 96-well plate (Thermo Fisher Scientific, Waltham, MA, USA), and 20.0 μL of a reagent consisting of TaqMan^TM^ Universal PCR Master Mix, no AmpErase^TM^ UNG (11.25 μL, Thermo Fisher Scientific, Waltham, MA, USA), TaqMan^®^ Gene Expression Assay (1.125 μL, COL1A1, COL1A2, or GAPDH mRNA, Thermo Fisher Scientific, Waltham, MA, USA), and nuclease-free water (7.65 μL) was added. The plate was sealed with MicroAmp^®^ Optical Adhesive Film (Thermo Fisher Scientific, Waltham, MA, USA) and centrifuged at 170 *g* and 4 °C for 15 s, and the samples were analyzed using a QuantStudio^TM^ 12K Flex real-time PCR system (Thermo Fisher Scientific, Waltham, MA, USA). Data were processed using QuantStudio^TM^ 12K Flex software V1.x (Thermo Fisher Scientific, Waltham, MA, USA). Relative COL1A1 and COL1A2 mRNA expression levels were calculated after normalization to GAPDH levels.

### 2.13. Statistical Analyses

Statistical significance was determined using Dunnett’s test, implemented in JMP Pro software ver. 14.0. *p* < 0.05 was considered statistically significant.

## 3. Results

### 3.1. γδ T Cell Contact-Dependent Regulation of Collagen Type I in Lung Fibroblasts

To prepare a large number of purified Vγ9Vδ2 T cells (also termed Vγ2Vδ2 T cells, depending on which nomenclature system is used; hereinafter referred to as γδ T cells), we stimulated PBMCs derived from a healthy donor with PTA, a prodrug of the nitrogen-containing bisphosphonate 2-(thiazole-2-ylamino)ethylidene-1,1-bisphosphonate (TA), and interleukin-2 (IL-2). The proportion of γδ T cells in PBMCs on Day 0 was 2.5% but increased to 98.7% after an 11-day expansion using PTA/IL-2. A similar pattern of γδ T cell expansion, from 3.1% to 98.7%, was observed in a patient with IPF (Figure S1a). Almost all PTA/IL-2-expanded γδ T cells expressed natural killer group 2; member D (NKG2D, also termed CD314), a C-type lectin receptor; and DNAX accessory molecule-1 (DNAM-1, also termed CD226), an immunoglobulin superfamily receptor, suggesting that γδ T cells exhibit effector functions similar to natural killer (NK) cells. However, the expression of Fas ligand (FasL, CD95L, or CD178) and TRAIL (or CD253) was not observed (Figure S1b). There were no differences in γδ T cell phenotypes between the healthy volunteer and the patient with IPF.

**Figure 1:**
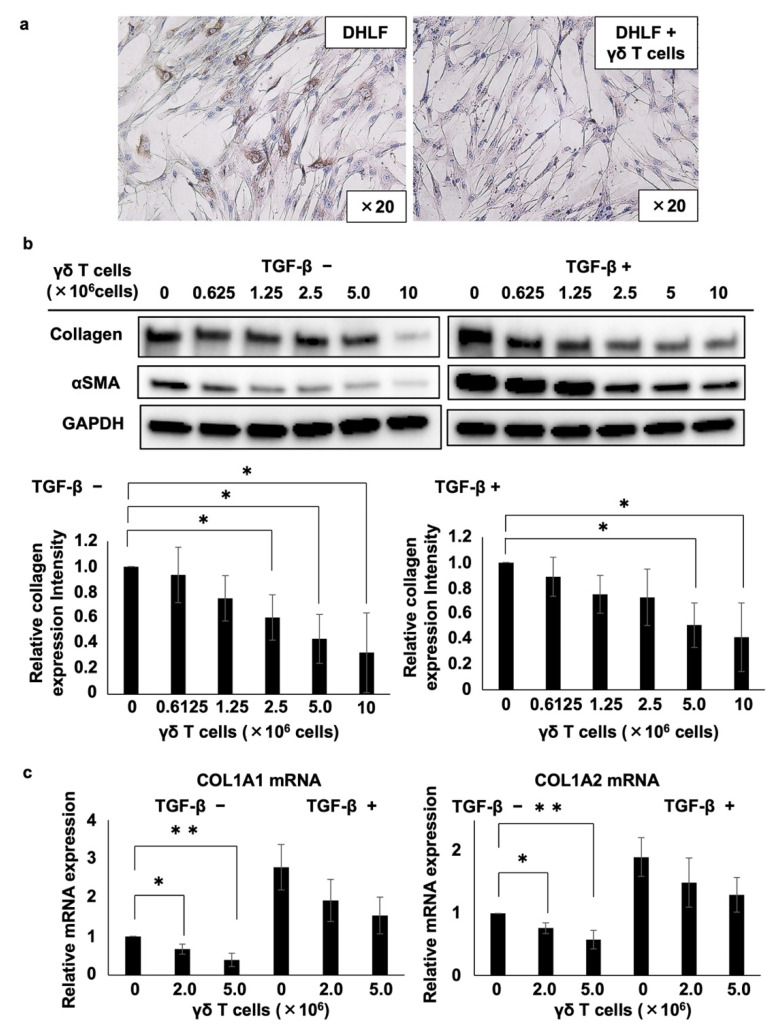
Effects of γδ T cells on lung fibroblasts. (**a**) Immunocytochemical analysis of the effects of γδ T cells on the expression of collagen type I in DHLF cells. DHLF cells (2.5 × 10^4^) were cultured overnight in a Lab-Tek chamber slide. PTA/IL-2-induced γδ T cells (2.0 × 10^6^) or RPMI 1640 medium (control) were then added. After co-culturing for two additional days, collagen type I in DHLF cells was stained and observed under a microscope. (**b**) Western blot analysis of collagen type I and αSMA in DHLF cells treated with Vγ2Vδ2 T cells. DHLF cells (1.5 × 10^5^) were cultured overnight in a 6-well plate in the presence (**right**) or absence (**left**) of 5 ng/mL TGF-β. The wells were then washed with PBS before adding 0, 0.625 × 10^6^, 1.25 × 10^6^, 2.5 × 10^6^, 5 × 10^6^, or 1 × 10^7^ PTA/IL-2-induced γδ T cells. After co-culturing for two additional days, the expression levels of collagen type I and αSMA in DHLF cells were evaluated via standard Western blot analysis. Relative collagen expression levels were calculated after normalization to GAPDH. (**c**) Real-time RT-PCR analysis of the expression of COL1A1 and COL1A2 mRNA in DHLF cells co-cultured with γδ T cells. DHLF cells (1.5 × 10^5^) treated with TGF-β (5 ng/mL) or control medium were co-cultured with 0, 2 × 10^6^, or 5 × 10^6^ PTA/IL-2-induced γδ T cells for 2 days under conditions similar to those for Western blot analysis. COL1A1 and COL1A2 mRNA expression in the DHLF cells was analyzed using real-time RT-PCR. Data are presented as mean ± standard deviation and are representatives of four independent experiments. Statistical significance was measured using Dunnett’s test (* *p* < 0.05, ** *p* < 0.01).

Then, we examined the effect of PTA/IL-2-induced γδ T cells on the expression of collagen type I in DHLF, a diseased human lung fibroblast cell line. DHLF cells were incubated in the presence of γδ T cells for 2 days, and the collagen levels were measured via immunocytochemistry. γδ T cells significantly reduced the expression of collagen type I in DHLF cells (Figure 1).

We also examined the effect of γδ T cells and transforming growth factor-β (TGF-β), an inducer of collagen type I, on the viability of DHLF cells. DHLF cell viability was determined via the standard luciferase-based assay. TGF-β and γδ T cells influenced the viability of DHLF cells (Figure S2), indicating that the reduction in collagen type I was not due to the cytotoxic effect of γδ T cells against DHLF cells.

Furthermore, we quantified the effect of γδ T cells on collagen type I in DHLF cells. After the DHLF cells were incubated for 2 days with serially diluted γδ T cells, the expression levels of collagen type I, α-smooth muscle actin (αSMA), and glyceraldehyde-3-phosphate dehydrogenase (GAPDH, as an internal control) were determined using standard Western blot assay. As depicted in the left panels of Figure 1b, γδ T cells downregulated the expression of both collagen type I and αSMA, a marker of myofibroblasts involved in the pathology of fibrotic conditions, depending on the number of γδ T cells, suggesting that γδ T cells can potentially ameliorate the symptoms of fibrotic diseases. Even when DHLF cells were pretreated with TGF-β, which led to the enhanced expression of collagen type I and αSMA, γδ T cells reduced their expression depending on the number of γδ T cells (right panels of Figure 1b). Moreover, γδ T cells derived from a patient with IPF also inhibited the expression of collagen type I and αSMA in DHLF cells (Figure S3).

The effect of γδ T cells on collagen mRNA expression was analyzed using RT-PCR. When DHLF cells were co-cultured with γδ T cells, both COL1A1 and COL1A2 mRNA expression levels were substantially downregulated in a γδ T cell number-dependent manner (Figure 1c). When DHLF cells were pretreated with TGF-β, γδ T cells suppressed the mRNA levels, although the inhibition was not statistically significant, possibly due to the greater mRNA induction in DHLF cells by TGF-β.

**Figure 2:**
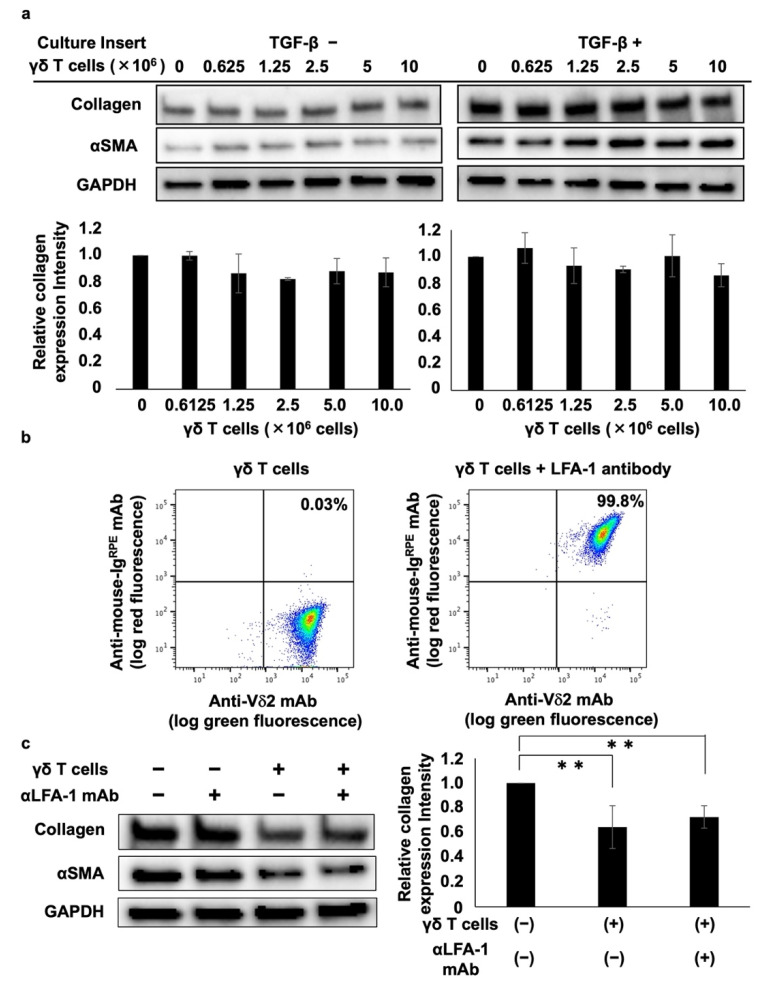
Cell–cell contact-dependent inhibition of collagen levels in lung fibroblasts by γδ T cells. (**a**) Effect of soluble factors derived from Vγ2Vδ2 T cells on collagen levels in lung fibroblasts. DHLF cells (1.5 × 10^5^) were seeded in a 6-well plate and incubated overnight in the presence or absence of 5 ng/mL TGF-β. The cells were then washed with PBS (-) and culture membrane inserts attached to the wells before adding serial dilutions of γδ T cells (0, 0.625 × 10^6^, 1.25 × 10^6^, 2.5 × 10^6^, 5 × 10^6^, 10 × 10^6^ cells). After incubation for 2 days, DHLF cells were dissolved in lysis buffer and collagen I and αSMA levels were determined via Western blot analysis. Relative collagen expression was measured after normalization to GAPDH expression levels. (**b**) Flow cytometric analysis of LFA-1 expression on γδ T cells. PTA/IL-2-induced γδ T cells were treated with 2 μg/mL of anti-LFA1 mouse antibody for 30 min. After washing with PBS (-), the cells were placed on ice and stained with FITC-conjugated anti-Vδ2 mAb and R-phycoerythrin-conjugated anti-mouse Ig for 15 min. The stained cells were washed three times with PBS (-) and analyzed using a FACSLyric flow cytometer. (**c**) Effect of anti-LFA-1 mAb on γδ T cell-mediated inhibition of collagen in lung fibroblasts. DHLF cells (1.5 × 10^5^) were cultured overnight and incubated for 2 days with γδ T cells (5 × 10^6^) in the presence or absence of anti-LFA-1 mAb. The expression levels of collagen I and αSMA in DHLF were examined via Western blot analysis. Data are presented as mean ± standard deviation and are representatives of three independent experiments. Statistical significance was measured using Dunnett’s test (** *p* < 0.01).

A trans-well culture system was used to analyze the mechanism underlying the γδ T cell-mediated inhibition of collagen type I. Analysis of the effect of PTA/IL-2-induced γδ T cells on collagen and αSMA protein expression showed that γδ T cells did not suppress collagen type I or αSMA expression, regardless of the presence or absence of TGF-β (Figure 2a), suggesting that the γδ T cell-mediated inhibition of collagen and αSMA is dependent on γδ T cell–DHLF cell contact.

We also examined the effect of lymphocyte function-associated antigen-1 (LFA-1) on γδ T cell-mediated inhibition because LFA-1 is highly expressed on PTA/IL-2-induced γδ T cells (Figure 2b) and is a member of the integrin superfamily of adhesion molecules that interacts with intercellular adhesion molecule 1 (ICAM-1) expressed on fibroblasts and vascular endothelial cells. Incubating DHLF and γδ T cells with anti-LFA-1 neutralizing mAb showed that γδ T cells’ suppression of collagen type I and αSMA was not reversed by the neutralizing mAb (Figure 2c), indicating that the γδ T cell-mediated suppression of collagen type I and αSMA does not occur through LFA-1–ICAM-1 interaction.

### 3.2. Effect of PTA/IL-2-Expanded γδ T Cells on Lung Fibroblasts in the Presence of HMBPP

**Figure 3:**
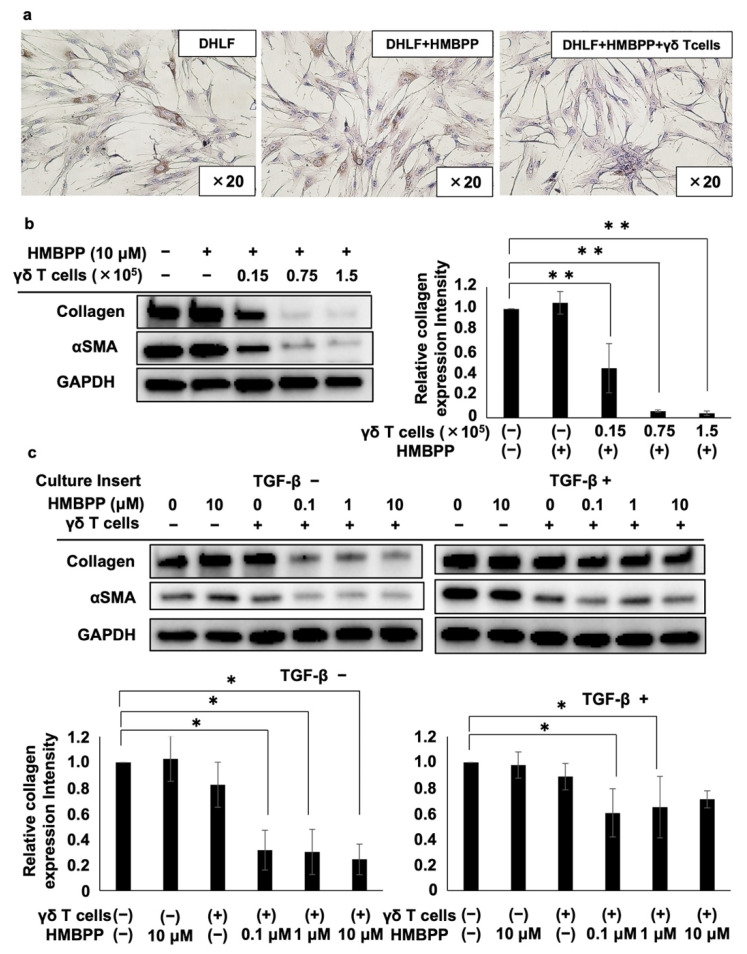
Effect of HMBPP on γδ T cell-mediated suppression of collagen in lung fibroblasts. (**a**) Immunocytochemical analysis of collagen type I in lung fibroblasts challenged with HMBPP-activated γδ T cells. DHLF cells (2.5 × 10^4^) were incubated overnight in a Lab-Tek chamber slide. γδ T cells (5.0 × 10^3^) or RPMI 1640 medium in the presence or absence of 10 μM HMBPP were then added. The slide was incubated for 2 days, and collagen type I levels in DHLF cells were then measured. (**b**) Western blot analysis of collagen type I and αSMA in lung fibroblasts challenged with HMBPP-activated γδ T cells. DHLF cells (1.5 × 10^5^) were cultured overnight in a 6-well plate. γδ T cells (0, 0.15 × 10^5^, 0.75 × 10^5^, or 1.5 × 10^5^) in the presence or absence of HMBPP (10 μM) were then added. After co-culturing for 2 days, collagen type I and αSMA protein levels in DHLF cells were determined via Western blot analysis. (**c**) Western blot analysis of collagen type I and αSMA in TGF-β-pretreated lung fibroblasts challenged with HMBPP-activated γδ T cells through culture membrane inserts. DHLF cells (1.5 × 10^5^) were incubated overnight in the presence (right panel) or absence (left panel) of TGF-β (5 ng/mL). The cells were washed with PBS (-), and γδ T cells (5 × 10^6^) diluted serially with HMBPP (0, 0.1, 1, or 10 μM) were added through the culture membrane inserts. After incubation for 2 days, collagen type I and αSMA levels in DHLF cells were analyzed via Western blot analysis. Relative collagen expression was calculated by normalization to GAPDH. Data are presented as mean ± standard deviation and are representatives of three independent experiments. Statistical significance was determined using Dunnett’s test (* *p* < 0.05, ** *p* < 0.01).

We examined the effect of HMBPP, a pyrophosphomonoester γδ T cell antigen, on γδ T cell-mediated collagen type I suppression in human fibroblasts. When DHLF cells were co-cultured with PTA/IL-2-induced γδ T cells in the presence of HMBPP, even a small number of γδ T cells (as low as 5 × 10^3^) suppressed collagen type I expression in DHLF cells, whereas collagen type I levels were not altered by HMBPP in the absence of γδ T cells (Figure 3a), indicating that the activation of γδ T cells by HMBPP led to the effective suppression of collagen type I expression in lung fibroblasts.

To further examine the effect of HMBPP on γδ T cell-mediated collagen type I suppression, DHLF cells were cultured in serial dilutions of γδ T cells in the presence of HMBPP, and collagen type I and αSMA were monitored via Western blot analysis. Collagen type I expression was significantly reduced by HMBPP-activated γδ T cells in a γδ T cell number-dependent manner (left panel of Figure 3b). αSMA was also downregulated by γδ T cells in the presence of HMBPP. Quantification of Western blot bands showed the statistically significant suppression of collagen type I (Figure 3b) and αSMA (Figure S4) by HMBPP-activated γδ T cells.

Since HMBPP significantly enhanced the suppressive effect of PTA/IL-2-induced γδ T cells on collagen type I and αSMA expression in human fibroblast cells, we examined the effect of soluble factors secreted by HMBPP-stimulated γδ T cells on DHLF cells. When DHLF cells were cultured with γδ T cells in a trans-well plate in the presence of serial HMBPP dilutions, both collagen type I and αSMA levels were significantly reduced at HMBPP concentrations equal to or greater than 0.1 μM compared with those in cells cultured in the absence of HMBPP (left panels of Figure 3c). Similar results were obtained when TGF-β-treated DHLF cells were exposed to soluble factors derived from γδ T cells in the presence of serial HMBPP dilutions (right panels of Figure 3c), suggesting that soluble factors derived from HMBPP-stimulated γδ T cells demonstrate potent collagen type I-suppressive activity in DHLF cells.

### 3.3. Effect of Lymphokines Secreted from HMBPP-Stimulated γδ T Cells on Collagen Type I in Lung Fibroblasts

**Figure 4:**
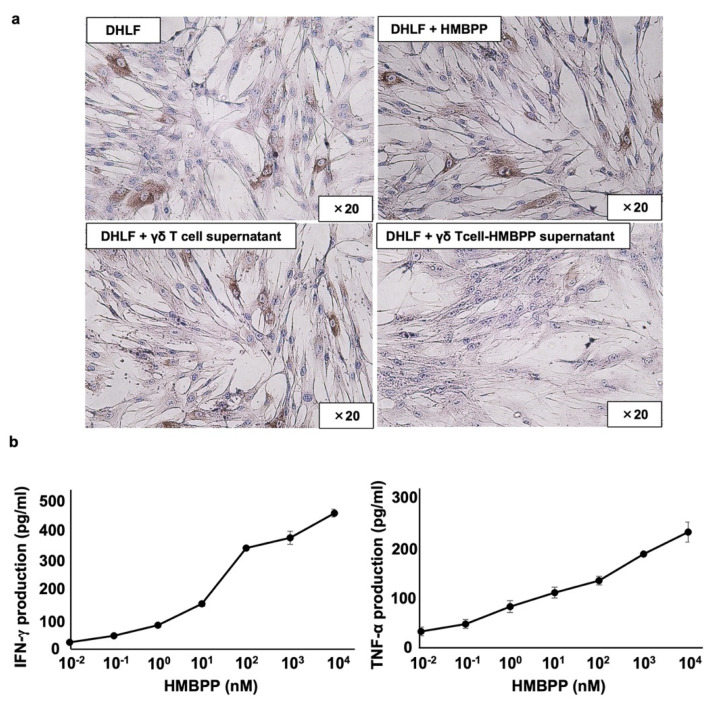
Effect of γδ T cell supernatant stimulated with HMBPP on collagen expression in lung fibroblasts. (**a**) Immunocytochemical analysis of collagen type I in lung fibroblasts treated with HMBPP-stimulated γδ T cell supernatant. DHLF cells (2.5 × 10^4^) were cultured overnight in a Lab-Tek chamber slide. After the supernatants were aspirated, 1 mL of RPMI 1640 medium and 1 mL of γδ T cell supernatant (5.0 × 10^6^ cells), cultured overnight in the presence or absence of HMBPP (10 μM), were added to the chamber slide. The cells were cultured for two additional days, and collagen type I expression in DHLF cells was analyzed via immunocytochemistry. (**b**) Effect of HMBPP on IFN-γ and TNF-α secreted by γδ T cells. γδ T cells (4 × 10^5^) were cultured with serial dilutions of HMBPP (0.01 nM, 0.1 nM, 1 nM, 10 nM, 100 nM, 1 μM, or 10 μM) in a 96-well flat-bottom plate. After incubation for 24 h, IFN-γ and TNF-α levels in the culture supernatants were analyzed using ELISA. Data are presented as mean ± standard deviation and are representatives of three independent experiments.

To analyze the mechanism underlying the suppression of collagen type I by supernatants from HMBPP-stimulated γδ T cells, DHLF cells were treated with γδ T cell culture supernatants incubated with or without HMBPP and analyzed for collagen type I expression via immunocytochemistry. Compared with that of unstimulated γδ T cells, culture supernatants of HMBPP-stimulated γδ T cells markedly suppressed collagen type I expression (Figure 4a).

Since γδ T cells secrete a variety of lymphokines in response to HMBPP, we measured interferon-γ (IFN-γ) and tumor necrosis factor-α (TNF-α) levels in culture supernatants of HMBPP-stimulated γδ T cells using ELISA. IFN-γ and TNF-α were secreted into culture media in an HMBPP concentration-dependent manner when PTA/IL-2-expanded γδ T cells were incubated with serial HMBPP dilutions (Figure 4b).

**Figure 5:**
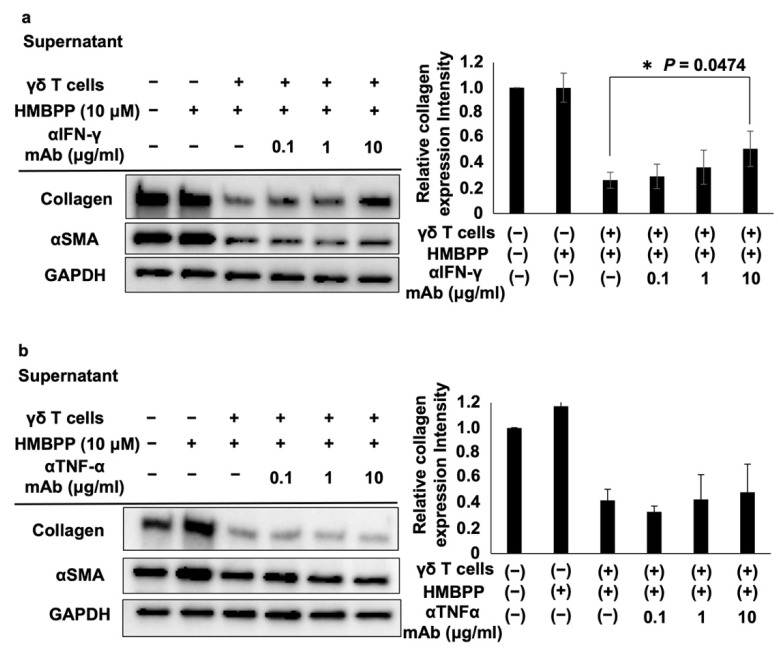
Effect of anti-IFN-γ and anti-TNF-α mAbs on collagen levels in DHLF cells treated with HMBPP-stimulated γδ T cell supernatants. Western blot analysis of collagen type I and αSMA in lung fibroblasts treated with HMBPP-stimulated γδ T cell supernatant and anti-IFN-γ antibody (**a**) or anti-TNFα antibody (**b**). DHLF cells (1.5 × 10^5^) were cultured overnight in a 6-well plate. After the supernatants were aspirated, culture supernatants (1 mL each) of HMBPP-stimulated γδ T cells and serial dilutions of anti-IFN-γ or anti-TNF-α mAb (0, 0.1 μg/mL, 1 μg/mL, or 10 μg/mL) were added to the wells, and the plate was incubated for two additional days. Collagen type I and αSMA levels in DHLF cells were analyzed via Western blot analysis. Relative collagen expression was calculated by normalization to GAPDH. Data are presented as mean ± standard deviation and are representatives of three independent experiments. Statistical significance was measured using Dunnett’s test (* *p* < 0.05).

The effects of these lymphokines on the collagen type I-suppressive effect of culture supernatants of HMBPP-stimulated γδ T cells were examined. When DHLF cells were treated with serial dilutions of anti-IFN-γ neutralizing mAb and challenged with culture supernatants of HMBPP-stimulated γδ T cells, the collagen type I-suppressive effect of the supernatant was partially reversed in an anti-IFN-γ mAb concentration-dependent manner (Figure 5a), demonstrating that IFN-γ plays an essential role in suppressing collagen in DHLF cells. However, anti-TNF-α neutralizing mAb failed to reverse this suppressive effect (Figure 5b).

Since human IFN-γ likely facilitates the collagen type I-suppressive effect through human IFN-γ receptors, we examined whether the culture supernatants of human γδ T cells reduce collagen levels in mouse fibroblasts that do not express human IFN-γ receptors. When the mouse lung fibroblast cell line Mlg was incubated with human γδ T cells in the presence of HMBPP in a trans-well culture system, collagen type I was not suppressed. The absence of a culture membrane insert allowed cell–cell contact between γδ T and Mlg cells; however, the statistically significant suppression of collagen was not observed, suggesting that the suppressive effect of collagen by human γδ T cells is species-specific (Figure S5).

### 3.4. Effect of IL-18 on Collagen Type I-Suppressive Effect of HMBPP-Stimulated γδ T Cell Culture Supernatants

**Figure 6:**
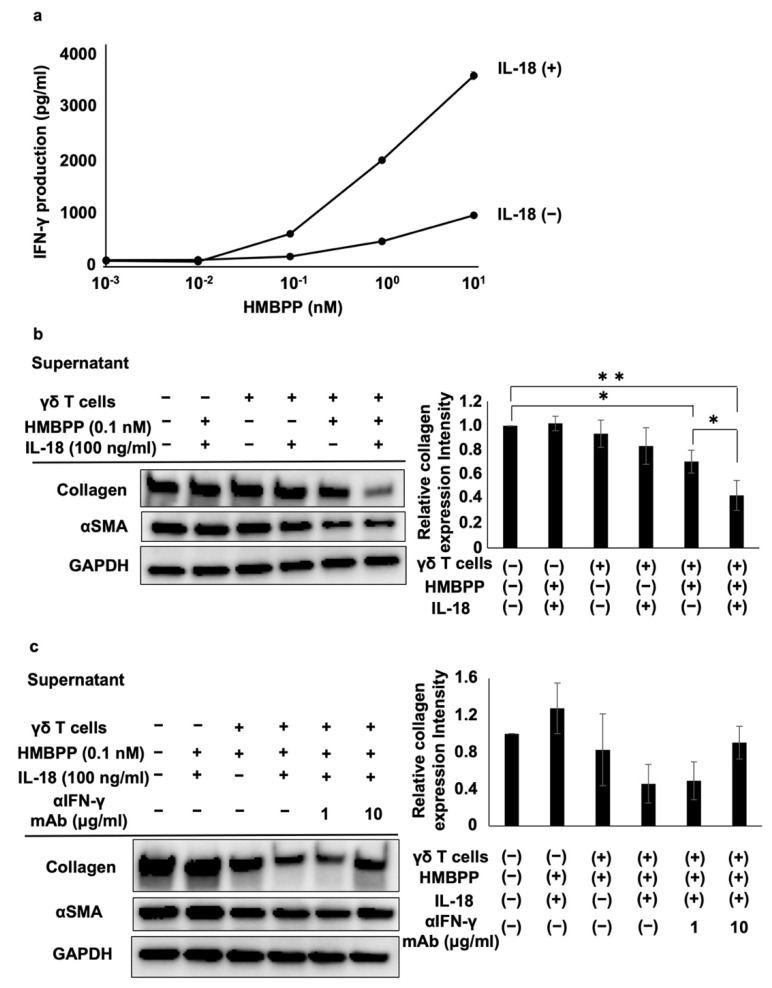
Effect of IL-18 on the suppression of collagen type I and αSMA in lung fibroblasts by HMBPP-stimulated γδ T cell supernatant. (**a**) Effect of IL-18 on IFN-γ secreted from HMBPP-activated γδ T cells. γδ T cells (4 × 10^5^) were stimulated for 24 h in a 96-well flat-bottom plate using serial dilutions of HMBPP (0.001, 0.01, 0.1, 1, or 10 nM) in the presence or absence of IL-18 (100 ng/mL). Supernatants were collected and IFN-γ levels were measured via ELISA. (**b**) Effect of IL-18 on collagen type I and αSMA levels in lung fibroblasts treated with HMBPP-stimulated γδ T cell culture supernatants. DHLF cells (1.5 × 10^5^) were cultured overnight in a 6-well plate. After supernatants were aspirated, culture supernatants (1 mL each) of γδ T cells treated with or without HMBPP (0.1 nM) in the presence or absence of IL-18 (100 ng/mL) were added to the wells. The plate was incubated for 2 days, and collagen type I and αSMA levels in DHLF cells were analyzed via Western blot analysis. Relative collagen expression was calculated by normalization to GAPDH. (**c**) Effect of anti-IFN-γ mAb on the suppression of collagen type I and αSMA in lung fibroblasts treated with HMBPP/IL-18-stimulated γδ T cell culture supernatants. DHLF cells (1.5 × 10^5^) were cultured overnight in a 6-well plate. After the supernatants were aspirated, anti-IFN-γ mAb (0, 1, or 10 μg/mL) and culture supernatants (1 mL) of γδ T cells incubated overnight with HMBPP (0.1 nM) and IL-18 (100 ng/mL) were added to the wells. The cells were cultured for two additional days, and collagen type I and αSMA in DHLF cells were analyzed via Western blot analysis. Relative collagen expression was calculated by normalization to GAPDH. Data are presented as mean ± standard deviation and are representatives of three independent experiments. Statistical significance was determined using Dunnett’s test (* *p* < 0.05, ** *p* < 0.01).

IL-18 enhances the ability of IL-12 to stimulate the secretion of IFN-γ from immune effector cells. Hence, we examined the effect of IL-18 on the secretion of IFN-γ from HMBPP-stimulated γδ T cells. When γδ T cells were stimulated with serial dilutions of HMBPP in the presence or absence of IL-18, γδ T cells secreted IFN-γ depending on the concentration of HMBPP, and lymphokine production was increased following the addition of IL-18 (Figure 6a).

To examine the influence of IL-18 on the suppressive effect of γδ T cell supernatants, PTA/IL-2-induced γδ T cells were re-stimulated with HMBPP in the presence or absence of IL-18, and DHLF cells were challenged with the culture supernatants. Collagen type I and αSMA were significantly reduced in human fibroblasts treated with culture supernatants of HMBPP/IL-18-stimulated γδ T cells (Figure 6b). Adding anti-IFN-γ neutralizing mAb reversed the suppressive effect of the supernatants (Figure 6c), indicating that IFN-γ in the culture supernatants plays a major role in the suppression of collagen type I.

## 4. Discussion

Several studies have reported the effect of human γδ T cells on the regulation of collagen type I in fibroblasts, although the results are contradictory [17,19,20,22], partially because the purity, quality, and number of γδ T cells were insufficient to appropriately evaluate these effects. In this study, we prepared a large number of purified human γδ T cells using PTA, a nitrogen-containing bisphosphonate prodrug that efficiently stimulates γδ T cells [24,25]. The purity of the PTA/IL-2-expanded γδ T cells used in this study was at least 98%, and the cells expanded for 11 days before γδ T cell effector functions deteriorated.

PTA is a highly hydrophobic compound that readily penetrates cell membranes. Once the compound is internalized by cells, it is hydrolyzed by intracellular esterases to give TA, a biologically active bisphosphonate that inhibits FDPS [23]. When FDPS is inhibited, the immediate upstream metabolites, IPP and DMAPP, accumulate in the cytoplasm and bind to the B30.2 intracellular domain of butyrophilin 3A1 (BTN3A1) [25,26]. Although the precise mechanism has not been elucidated, the B30.2/IPP/DMAPP complex activates γδ T cells in a BTN3A1/BTN2A1-dependent manner [27]. This protocol for expanding γδ T cells facilitated the examination of the role of γδ T cells in the suppression of collagen type I in lung fibroblasts.

PTA/IL-2-induced γδ T cells effectively reduced the quantities of collagen type I in lung fibroblasts, in a cell–cell contact-dependent manner, based on the results from a trans-well plate assay system. Therefore, we hypothesized that certain adhesion molecules may be involved in the γδ T cell-mediated suppression of collagen type I [28,29]. Several adhesion molecules that play a pivotal role in the adhesion of γδ T cells to fibroblasts are expressed on γδ T cell surfaces, including LFA-1, very late antigen-4 (VLA-4), and VLA-5 [30,31,32]. LFA-1 is expressed on IPP-stimulated γδ T cells and is activated following its ligation to its ligands, including ICAM-1 [31,32].

In this study, we demonstrated that PTA/IL-2-expanded γδ T cells expressed high LFA-1 levels. Treating γδ T cells with anti-LFA-1 neutralizing mAb, however, did not reverse the suppression of collagen type I expression, suggesting that the modulation of collagen type I in lung fibroblasts by γδ T cells is not mediated by LFA-1.

Since human γδ T cells exhibited TCR-independent cytotoxicity against human lung cancer cells [25], it is possible that the suppression of collagen type I expression by γδ T cells is caused by γδ T cell-mediated cytotoxic effects against lung fibroblasts. Based on the cytotoxicity assay results, however, γδ T cells showed no cytotoxicity against human lung fibroblasts under the conditions in which this study was conducted. Although γδ T cells did not alter the viability of lung fibroblasts, they significantly suppressed the expression of COL1A1 and COL1A2 mRNAs in lung fibroblasts, suggesting that these cells transduce signals in fibroblasts, inhibiting the transcription and translation of collagen type I genes, wherein the interaction between γδ T cells and fibroblasts plays an essential role in signal transduction. Cell–cell contact-dependent suppression of collagen type I was shown to be species-specific, suggesting the involvement of human-specific ligand-receptor combination in signal transduction.

The effect of activated γδ T cells on the regulation of collagen type I in lung fibroblasts was examined because activated γδ T cells express a variety of effector molecules that may mediate the suppression of collagen type I expression. We used HMBPP, a phosphoantigen, to activate the γδ T cells. This phosphoantigen is biosynthesized through the 2-C-methyl-D-erythritol 4-phosphate (MEP) pathway and is converted to IPP in bacteria, protozoa, and plants [33]. HMBPP binds B30.2 of BTN3A1-like IPP and induces effector functions in γδ T cells, including cytotoxicity against tumor cells and the secretion of proinflammatory cytokines and growth factors [33,34].

HMBPP-activated γδ T cells suppressed collagen type I levels in human lung fibroblasts more efficiently than unstimulated γδ T cells. Thus, we examined whether HMBPP-activated γδ T cells secrete soluble factors that might be involved in the suppression of collagen type I. Supernatants of HMBPP-activated γδ T cells suppressed collagen type I in lung fibroblasts, unlike unstimulated γδ T cells, suggesting that certain humoral factors secreted by HMBPP-activated γδ T cells are involved in collagen suppression. Since human γδ T cells can produce antifibrotic cytokines such as IFN-γ and TNF-α [35,36,37], we examined the effect of these cytokines secreted from HMBPP-activated γδ T cells. ELISA-based analysis using neutralizing mAbs showed that IFN-γ, but not TNF-α, was involved in the suppression of collagen type I in lung fibroblasts, although both cytokines previously demonstrated the ability to inhibit collagen synthesis [38,39,40], suggesting that IFN-γ plays a major role in the γδ T cell-mediated suppression of collagen type I in lung fibroblasts. Some in vitro studies have shown that IFN-γ inhibits collagen synthesis and type I procollagen mRNA levels in fibroblasts and suppresses fibroblast proliferation [40,41]. Furthermore, an in vivo study indicated that IFN-γ administration decreases fibroblast numbers and hydroxyproline content in a bleomycin-induced pulmonary fibrosis mouse model [42]. Therefore, IFN-γ is closely associated with lung fibroblasts, and our findings are consistent with the findings of these previous reports.

Since IL-18 is a member of the IL-1 cytokine superfamily, enhances IFN-γ production, and stimulates effector T cells together with IL-12, IL-2, or antigens, we examined the effect of IL-18 on the suppression of collagen type I in lung fibroblasts [43,44]. As expected, collagen type I expression was significantly suppressed by supernatants of HMBPP/IL-18-stimulated γδ T cells, even at low HMBPP concentrations. Adding anti-IFN-γ neutralizing mAb reversed the suppressive effect of the supernatants, suggesting that IFN-γ is a pivotal factor in the supernatants of HMBPP/IL-18-stimulated γδ T cells that suppresses collagen type I in lung fibroblasts.

Collagen type I levels in mouse fibroblasts were not altered by treatment with the supernatants of HMBPP/IL-18-stimulated human γδ T cells because human IFN-γ is not recognized by murine IFN-γ receptors. Although murine γδ T cells have antifibrotic properties [11,12,13,14,15,16], it is difficult to establish murine models for studying the suppression of collagen type I by HMBPP/IL-18-stimulated γδ T cells because mice lack an HMBPP-reactive γδ T cell equivalent [45]. Currently, efforts are being devoted to developing protocols for the adoptive transfer of human γδ T cells in the field of cancer immunotherapy, which is well tolerated [46].

Since antifibrotic therapies based on small-molecule drugs do not currently have satisfactory outcomes, it is imperative to develop alternative therapeutic modalities such as γδ T cell-based immunotherapies for lung fibrosis. Since our γδ T cell culture system based on PTA allows us to prepare a large number of highly purified human γδ T cells with no restriction to major histocompatibility complex class I or II, it may be possible to develop γδ T cell-based off-the-shelf drugs for lung fibrotic diseases.

There are some limitations to this study. First, the detailed mechanism underlying the direct inhibition of collagen in lung fibroblasts by γδ T cells is unknown. Although cell–cell contact is important for collagen suppression, additional studies are needed to examine cell-surface markers and pathways involved in collagen inhibition by γδ T cells. Second, there might be other humoral factors in the supernatant of γδ T cells that are associated with collagen inhibition. Although IFN-γ plays an important role in collagen inhibition by the supernatant of γδ T cells, the collagen-suppressive effect of the supernatant was not completely reversed by anti-IFN-γ mAb alone. This suggests that other humoral factors secreted by γδ T cells might also be related to collagen inhibition. Despite these limitations, this study provides considerable insight into the development of γδ T cell-based immunotherapies for lung fibrosis.

## 5. Conclusions

In summary, we demonstrated that collagen type I expression in lung fibroblasts can be suppressed by γδ T cells through two distinct mechanisms: a cell–cell contact-dependent pathway and an IFN-γ-mediated pathway. Γδ-TCRs are not involved in the former mechanism and, thus, cell–cell contact-dependent therapy is expected to be mild and non-invasive. In contrast, the latter mechanism is mediated by proinflammatory cytokines, and TCR-mediated cell therapy can be invasive but effective. Nevertheless, further elucidation of the mechanism underlying the γδ T cell-mediated suppression of collagen type I in lung fibroblasts is necessary.

## Data Availability

The datasets generated during and/or analyzed during the current study are available from the corresponding author on reasonable request.

## Supplementary Materials

The following supporting information can be downloaded at: https://www.mdpi.com/article/10.3390/cells11182816/s1, Figure S1: Flow cytometric analysis and PTA/IL-2-induced expansion of peripheral blood γδ T cells; Figure S2: Effect of γδ T cells on the viability of DHLF cells in the presence or absence of TGF-β; Figure S3: Effect of γδ T cells derived from a patient with IPF on the expression of collagen type I and αSMA in lung fibroblasts; Figure S4: Effect of γδ T cells on αSMA levels in human fibroblasts in the presence of HMBPP; Figure S5: Western blot analysis of collagen type I in mouse lung fibroblasts co-cultured with human γδ T cells
