# Supplementary material for: Two Distinct Mechanisms Underlying γδ T Cell-Mediated Regulation of Collagen Type I in Lung Fibroblasts
Source: Cells. 2022 Sep 9;11(18):2816. doi: 10.3390/cells11182816 (PMC9496746; doi:10.3390/cells11182816)
Supplement: Supplementary file 1 [file cells-11-02816-s001.zip › cells-1880313-supplementary.pdf]

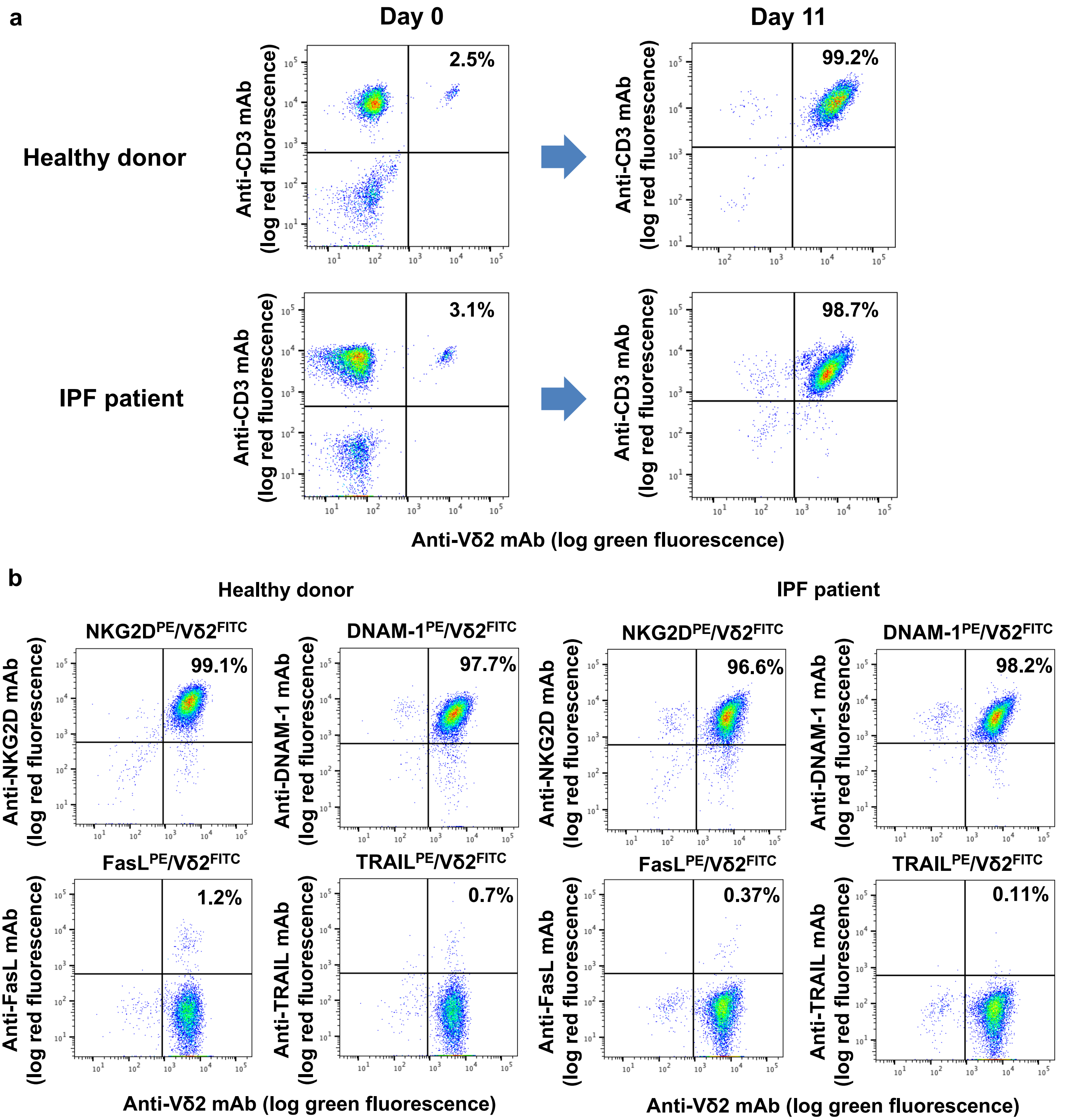

Supplementary Figure 1. Flow cytometric analysis and PTA/IL-2-induced expansion of peripheral blood γδ T cells. (A) Flow cytometric analysis of γδ T cells derived from a healthy donor and a patient with IPF before and after expansion with PTA/IL-2. PBMC derived from a healthy adult volunteer or a patient with IPF were stimulated with 1μM of PTA in Yssel's medium supplemented with human AB serum and γδ T cells were expanded with IL-2 for 11 days. On days 0 and 11, the expanded cells were stained with PE-conjugated anti-CD3 mAb and FITC-conjugated anti-Vδ2 mAb and analyzed through a FACS Lyric flow cytometer. **(B) Phenotypic analysis of PTA/IL-2-induced γδ T cells derived from a healthy donor and an IPF patient (B).** PTA/IL-2-induced γδ T cells derived from a healthy donor or an IPF patient were stained with PE-conjugated anti-NKG2D, DNAM-1, FasL or TRAIL mAb and FITC-conjugated anti-Vδ2 mAb and analyzed through a FACS Lyric flow cytometer.

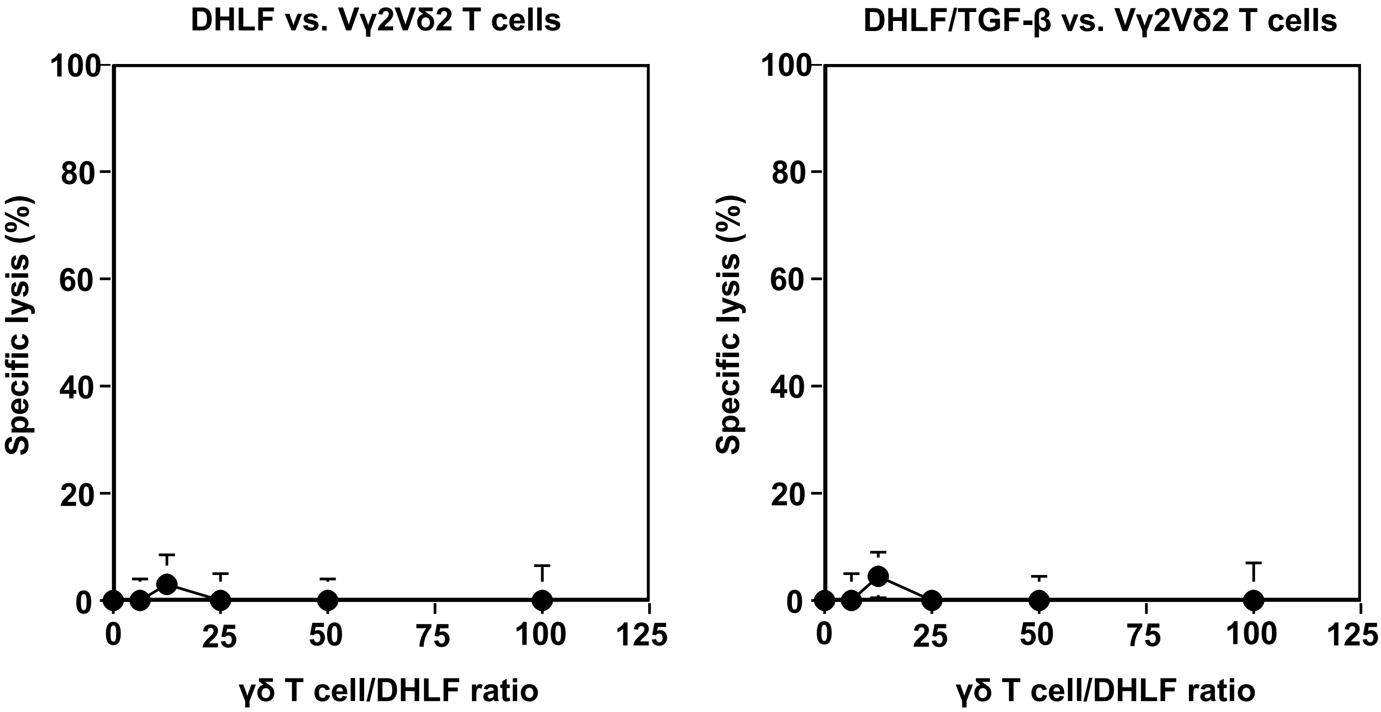

Supplementary Figure 2. Effect of γδ T cells on the viability of DHLF cells in the presence or absence of TGF-β. DHLF cells (2×10^4 cells) were incubated in the presence (right) or absence (left) of 5 ng/mL of TGF-β overnight, to which were added PTA/IL-2-induced γδ T cells at γδ T cell/DHLF ratios of 0:1, 6.25:1, 12.5:1, 25:1, 50:1 and 100:1. After incubation for 24 more hr, the γδ T cells were removed and the amount of adenosine triphosphate in viable DHLF cells was quantified by the standard luciferase assay. The specific lysis (%) was calculated as 100-(100 x experimental luminescence/control luminescence).

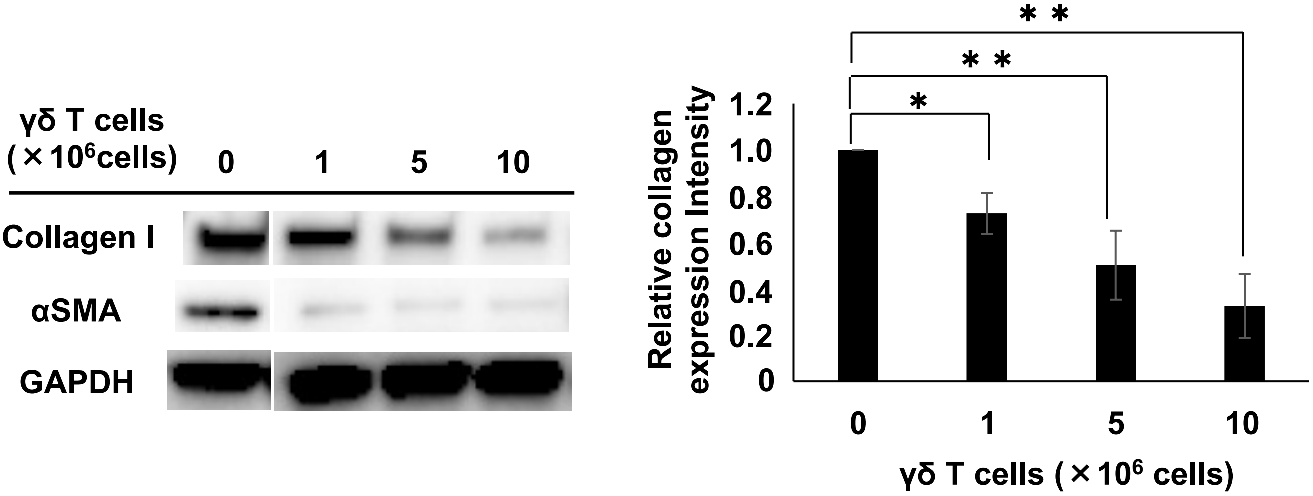

Supplementary Figure 3. Effect of γδ T cells derived from a patient with IPF on the expression of collagen type I and αSMA in lung fibroblasts. After DHLF cells (1.5 $\times 10^5$ cells) were cultured in a 6 well plate overnight, γδ T cells derived from an IPF patient were added to the wells (0, 1.0 $\times 10^6$, 5.0 $\times 10^6$ or 10 $\times 10^6$ cells per well) and the plate was incubated for 2 additional days. The expression of collagen type I and αSMA proteins in DHLF was analyzed through Western blotting analyses. Relative collagen expression levels were normalized to GAPDH. Data are presented as mean values ± standard deviations and are representative of three independent experiments. Dunnet test was employed for statical analyses (* $p<0.05$, ** $p<0.01$).

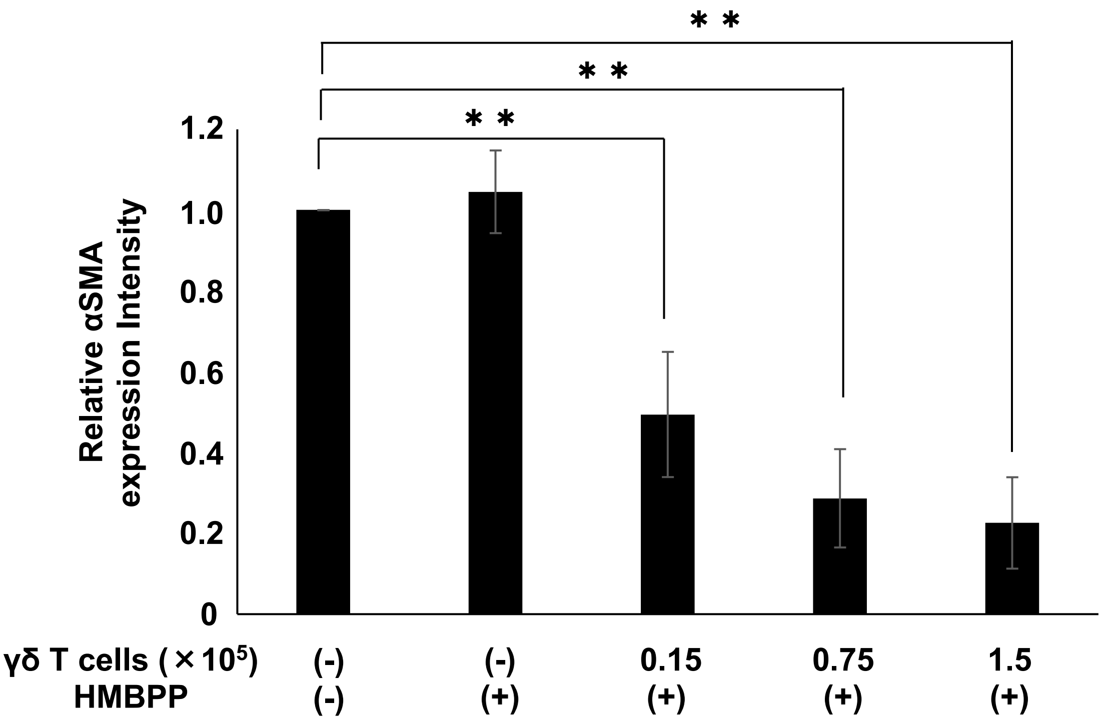

Supplementary Figure 4. Effect of γδ T cells on αSMA levels in human fibroblasts in the presence of HMBPP. DHLF cells (1.5×10^5 cells) were incubated in a 6 well plate overnight and the fibroblast cells were incubated with PTA/IL-2-induced γδ T cells (0, 0.15×10^5, 0.75×10^5, or 1.5×10^5 cells) in the presence or absence of HMBPP (10 μM). After co-culture for 2 additional days, αSMA levels in DHLF were quantified through Western blot analyses. αSMA expression was normalized to GAPDH to determine relative expression levels. Data are presented as mean values ± standard deviations. Dunnet test was employed for statistical analyses (* $p<0.05$, ** $p<0.01$).

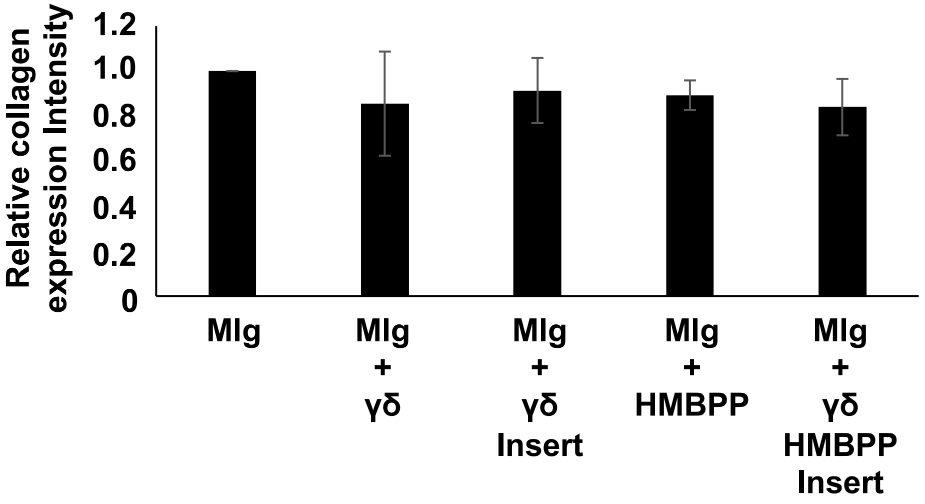

Supplementary Figure 5. Western blot analysis of collagen type I in mouse lung fibroblasts co-cultured with human γδ T cells. Mlg cells (1.5×10^5 cells) in a 6-well plate were prepared in the same way as described for DHLF cells. Human γδ T cells (5×10^6 cells) were added to the wells directly or through culture membrane inserts, to which were added HMBPP (10 μM) or medium. The plate was cultured for 2 additional days and Mlg cells were examined for collagen expression through Western blotting. Relative collagen expression was normalized to GAPDH to determine relative expression levels. Data are presented as mean values ± standard deviations. Dunnet test was employed for statistical analyses (* $p<0.05$, ** $p<0.01$).
